# Associations of endogenous estrogens, plasma Alzheimer’s disease biomarkers, and *APOE4* carrier status on regional brain volumes in postmenopausal women

**DOI:** 10.3389/fnagi.2024.1426070

**Published:** 2024-07-09

**Authors:** Katrina A. Wugalter, Rachel A. Schroeder, Rebecca C. Thurston, Minjie Wu, Howard J. Aizenstein, Ann D. Cohen, M. Ilyas Kamboh, Thomas K. Karikari, Carol A. Derby, Pauline M. Maki

**Affiliations:** ^1^Department of Psychology, University of Illinois Chicago, Chicago, IL, United States; ^2^Departments of Psychiatry, Epidemiology, Psychology, and Clinical and Translational Science, University of Pittsburgh, Pittsburgh, PA, United States; ^3^Department of Psychiatry, University of Pittsburgh, Pittsburgh, PA, United States; ^4^Department of Psychiatry, University of Pittsburgh, Pittsburgh, PA, United States; ^5^Departments of Psychiatry, Human Genetics, and Epidemiology, University of Pittsburgh, Pittsburgh, PA, United States; ^6^The Saul R. Korey Department of Neurology, Department of Epidemiology & Population Health, Albert Einstein College of Medicine, Bronx, NY, United States; ^7^Departments of Psychiatry, Psychology and Obstetrics & Gynecology, University of Illinois Chicago, Chicago, IL, United States

**Keywords:** brain volume, postmenopause, endogenous estrogens, *APOE4*, amyloid, tau

## Abstract

**Background:**

Women carrying the *APOE4* allele are at greater risk of developing Alzheimer’s disease (AD) from ages 65–75 years compared to men. To better understand the elevated risk conferred by *APOE4* carrier status among midlife women, we investigated the separate and interactive associations of endogenous estrogens, plasma AD biomarkers, and *APOE4* carrier status on regional brain volumes in a sample of late midlife postmenopausal women.

**Methods:**

Participants were enrolled in MsBrain, a cohort study of postmenopausal women (*n* = 171, mean age = 59.4 years, mean MoCA score = 26.9; race = 83.2% white, *APOE4* carriers = 40). Serum estrone (E1) and estradiol (E2) levels were assessed using liquid chromatography–tandem mass spectrometry. APOE genotype was determined using TaqMan SNP genotyping assays. Plasma AD biomarkers were measured using single molecule array technology. Cortical volume was measured and segmented by FreeSurfer software using individual T1w MPRAGE images. Multiple linear regression models were conducted to determine whether separate and interactive associations between endogenous estrogen levels, plasma AD biomarkers (Aβ42/Aβ40, Aβ42/p-tau181), and *APOE4* carrier status predict regional brain volume (21 regions per hemisphere, selected *a priori*); and, whether significant interactive associations between estrogens and AD biomarkers on brain volume differed by *APOE4* carrier status.

**Results:**

There was no main effect of *APOE4* carrier status on regional brain volumes, endogenous estrogen levels, or plasma AD biomarkers. Estrogens did not associate with regional brain volumes, except for positive associations with left caudal middle frontal gyrus and fusiform volumes. The interactive association of estrogens and *APOE4* carrier status on brain volume was not significant for any region. The interactive association of estrogens and plasma AD biomarkers predicted brain volume of several regions. Higher E1 and E2 were more strongly associated with greater regional brain volumes among women with a poorer AD biomarker profile (lower Aβ42/40, lower Aβ42/p-tau181 ratios). In *APOE4*-stratified analyses, these interactions were driven by non-*APOE4* carriers.

**Conclusion:**

We demonstrate that the brain volumes of postmenopausal women with poorer AD biomarker profiles benefit most from higher endogenous estrogen levels. These findings are driven by non-*APOE4* carriers, suggesting that *APOE4* carriers may be insensitive to the favorable effects of estrogens on brain volume in the postmenopause.

## 1 Introduction

Women comprise two-thirds of individuals living with Alzheimer’s disease (AD) in America (Alzheimer’s Association, 2023). In women, *APOE4* genotype is a stronger risk factor for developing AD from ages 65 to 75 years compared to men and confers disproportionate adverse effects on AD biomarkers at each disease stage (Neu et al., 2017; Sundermann et al., 2018). In studies of adults aged 70 years or older, *APOE4-*related adverse effects on default mode network connectivity, total tau levels, brain metabolism, and cortical volume were more pronounced in women than men (Damoiseaux et al., 2012; Sampedro et al., 2015). Women carrying the *APOE4* allele also had greater spread of neurofibrillary tangles and higher amyloid deposition at ages 60–75 compared to men, as identified postmortem (Corder et al., 2004). In contrast, some findings among older *APOE4* carriers demonstrate worse brain outcomes for men (Sundermann et al., 2018). Less well-known are the factors impacting brain health for *APOE4*+ women at midlife, when preventative efforts are most effective.

The greater vulnerability to *APOE4* effects in women has been attributed in part to the direct and interactive effects of estrogens on the brain (Valencia-Olvera et al., 2023). In animal models, estradiol (E2) influences the severity of AD biomarkers by preventing amyloid accumulation (Xu et al., 1998; Nilsen et al., 2006; Amtul et al., 2010; Kim et al., 2022) and tau hyperphosphorylation (Alvarez-De-La-Rosa et al., 2005). Furthermore, the effects of E2 on amyloid and tau have been shown to vary with *APOE4* carrier status in rodent and human studies (Kunzler et al., 2014; Kantarci et al., 2016a; Depypere et al., 2023). Administration of E2 increased amyloid deposition in the hippocampus and cortex in *APOE4* carrier mice only, indicating a vulnerability to E2 with the *APOE4* genotype (Kunzler et al., 2014). Subsequent research showed that *APOE* 3/3 and 3/4 mice had enhanced spatial memory and increased CA1 apical spine density after E2 administration, but these effects were not observed in *APOE* 4/4 mice indicating that *APOE4* homozygosity may impede the potential benefits of estrogens on the brain (Taxier et al., 2022). Human studies suggest that E2 may be either neutral or beneficial for *APOE4*+ women. In a large (*n* = 693) randomized clinical trial of menopausal hormone therapy (MHT) in early and late postmenopausal women, MHT had neutral effects on cognition regardless of *APOE4* genotype (Gleason et al., 2014). However, in a subset of women (*n* = 68) from that trial, MHT lowered Aβ deposition compared to placebo only in *APOE4*+ women (Kantarci et al., 2016a). Additionally, in an observational study examining 6-month change in plasma AD biomarkers in women before and after treatment with MHT (*n* = 193) in comparison to non-treated controls (*n* = 31), there were no overall differences in biomarkers between control and MHT groups, but group differences emerged when considering *APOE4* genotype (Depypere et al., 2023). Specifically, *APOE4*+ women who did not take MHT had worse AD biomarker outcomes (i.e., greater reduction in Aβ-42/p-tau 231 ratio) over time than women who took MHT. Further, among those in the MHT group, Aβ1-42 concentrations were higher (better) in *APOE4*+ women compared to *APOE4*− women. Thus, there is some consistency from basic and clinical science studies that *APOE* ε4 genotype might modify the effect of supplemental exogenous E2 on AD biomarkers, with greater benefits observed among *APOE4*+ women. It is yet unknown whether the effect of endogenous estrogens on the brain varies with *APOE* genotype.

In the postmenopause, women have low levels of endogenous E2 and estrone (E1) due to the cessation of ovarian steroid production (Randolph et al., 2011; Kim et al., 2017). While E2 levels are low overall, there are individual differences in trajectories of change of endogenous E2 across the menopause transition and into the postmenopause (Tepper et al., 2012). Trajectories include slow decline, flat slope, rise/slow decline, and rise/steep decline, associating differentially with biological and sociodemographic factors such as obesity, race, and ethnicity. Furthermore, endogenous E2 levels in the postmenopause have been associated with neuroimaging measures, such as resting state functional connectivity, particularly parahippocampal functional connectivity (Testo et al., 2024).

To better understand whether *APOE4*+ women are differentially sensitive to estrogen loss in the postmenopause, we investigated the separate and interactive associations of estrogens, plasma AD biomarkers, and *APOE4* carrier status on regional brain volumes in a sample of late midlife postmenopausal women. We were particularly interested in whether the interactive effects of endogenous estrogens and plasma AD biomarkers on brain volume vary by *APOE4* carrier status.

## 2 Materials and methods

### 2.1 Participants

Participants were enrolled in MsBrain, a cohort study of menopause and brain aging initiated in 2017 in Pittsburgh, PA, United States (Thurston et al., 2023). The MsBrain cohort (*N* = 274) is recruited from two sources: 170 participants previously (2015–2018) partook in MsHeart, a cross-sectional study of menopausal vasomotor symptoms (VMS) and cardiovascular health (Thurston et al., 2016), and 104 participants were recruited from the wider Pittsburgh community. Exclusion criteria in MsHeart included: current smoking; reported history of cardiovascular disease/stroke/cerebrovascular accident; insulin-dependent diabetes; Parkinson’s disease; hysterectomy and/or bilateral oophorectomy; current pregnancy; and use of HT (oral or transdermal estrogen and/or progesterone), select cardiovascular medications (beta blockers, calcium channel blockers, alpha-2 adrenergic agonists), selective estrogen receptor modulators (SERMS), aromatase inhibitors (AI), selective serotonin reuptake inhibitors (SSRIs) or serotonin norepinephrine reuptake inhibitors (SNRIs). MsBrain exclusion criteria additionally included: a reported history of dementia; seizure disorder; brain tumor; Parkinson’s disease; a history of head trauma with loss of consciousness; contraindications to MRI (e.g., metal in the body); current chemotherapy; active substance use; and pregnancy. In total, 238 women completed neuroimaging. Of those women, 21 were excluded due to incidental findings, seven learned English as a second language, four were missing hormone data, and three were perimenopausal. Given the neuroprotective effects of the *APOE2* allele (see Li et al., 2020), an additional 32 women who possessed the *APOE2* allele were excluded, yielding a final sample of 171 women.

### 2.2 Design

At the first visit, demographic and medical history were obtained. Body mass index (BMI) was calculated (kg/m^2^) from weight and height measured using a digital scale and a fixed stadiometer. Menopause status was determined using STRAW+10 criteria (Harlow et al., 2012). Three days after visit one, participants returned for a second visit wherein they underwent a blood draw and completed a one-hour cognitive test battery. During the cognitive testing, participants completed the Montreal Cognitive Assessment (MoCA) which is a brief screening instrument designed to identify mild cognitive impairment (Nasreddine et al., 2005). A MoCA score below 26 of 30 possible points indicates possible mild cognitive impairment (Nasreddine et al., 2005). At a third visit, on average 12 days after visit one, participants completed a neuroimaging protocol. Study procedures were approved by the University of Pittsburgh Human Research Protection Office. All participants provided written informed consent.

#### 2.2.1 Phlebotomy

Phlebotomy was performed after an eight-hour overnight fast. Blood was processed onsite with a Fisher Scientific Sorvall ST16R centrifuge and frozen in a − 80°C freezer until transportation to labs for assays.

##### 2.2.1.1 Estrogen

Serum E1 and E2 were assessed via liquid chromatography–tandem mass spectrometry at the University of Pittsburgh’s Small Biomarker Core, with inter- and intra-assay coefficients of variation of 5.0 and 8.1%, respectively. The lower limit of detection was 1.0 pg/mL for both estrogens. This method is sensitive to endogenous estrogen levels in the postmenopause (Nelson et al., 2004).

##### 2.2.1.2 *APOE* genotype

Genomic DNA was isolated from leukocytes using DNA purification kit (Qiagen, Valencia, CA). Genotypes for two *APOE* single-nucleotide polymorphisms (SNPs), rs429358 (E4) and rs7412 (E2), were determined by TaqMan SNP genotyping assays on ABI Prism 7900HT Sequence Detection System (Life Technologies, Grand Island, NY) as described elsewhere (Fan et al., 2022). Ten percent of samples were randomly selected and included as duplicates in genotyping run to estimate the assay error rate. The genotype outputs were converted to the six *APOE* genotypes: 2/2, 2/3, 2/4, 3/3, 3/4, and 4/4. After excluding E2 carriers*, APOE* status was categorized into *APOE4*+ (3/4 and 4/4) or *APOE4*− (3/3).

##### 2.2.1.3 Blood-based AD biomarkers

Plasma biomarker concentrations of Aβ42, Aβ40, and p-tau 181 were measured using Single molecule array (SIMOA) technology on an HD-X instrument (Quanterix). Frozen samples underwent a single thawing cycle. Plasma Aβ42 and Aβ40 were measured using the Neurology 4-Plex E (#103670) and p-tau181 was measured with the p-tau181 V2 Advantage (#103714) commercial assays from Quanterix. For each assay, quality control samples of different concentrations were analyzed in duplicates to estimate reproducibility. The pooled quality control data showed that the within- and between-run variations were approximately 10% for most assays. The present study examined Aβ42/40 and Aβ42/p-tau181 ratios due to their associations with brain Aβ and tau pathology (Pérez-Grijalba et al., 2019; Karikari et al., 2020) and to compare findings with previous work (Depypere et al., 2023). Lower ratios of these biomarkers of interest are considered more severe, as they indicate greater risk of future AD brain pathology (i.e., amyloid plaques and neurofibrillary tangles; Pérez-Grijalba et al., 2019; Karikari et al., 2020).

#### 2.2.2 Neuroimaging

All participants underwent neuroimaging at the MR Research Center of the University of Pittsburgh on a 3 T Siemens Tim Trio MR scanner, with a Siemens 64-channel head coil. Brain imaging assessments were performed MR pulse sequences were optimized for the multi-channel coil and follow the Human Connectome Project protocol. Structural data was acquired using a T1-weighted 3D Magnetization-prepared rapid gradient echo (MPRAGE: TR/TI/FA = 2300/900/9°, voxel size = 1 mm × 1 mm × 1 mm, Grappa 2). Regional brain volumes were divided by estimated total intracranial volume.

### 2.3 Analyses

#### 2.3.1 *A priori* regional brain volumes

There are 68 individual regions of interest (ROIs) included in the DK atlas. We refined the atlas to focus on 21 ROIs in regions with a high density of estrogen receptors and/or estrogen signaling, as determined by previous literature (see Table 1). We selected 13 frontal ROIs and 8 temporal ROIs in each hemisphere for a total of 42 ROIs.

**Table 1:** Regions of interest and their corresponding estrogen signaling.

DK Atlas Region	Estrogen reference
**Frontal lobe**	
	ER-alpha and ER-beta distribution in postmortem human brain (Österlund et al., 2000; Osterlund and Hurd, 2001) Reviews of estrogen signaling during the menopause transition (Brinton et al., 2015), in cholinergic (Newhouse and Dumas, 2015), glutamatergic, GABAergic, dopaminergic, and serotonergic pathways (Barth et al., 2015)
Superior frontal	ERs in AD patients postmortem (Kelly et al., 2008)
Rostral middle frontal gyrus	
Caudal middle frontal gyrus	
Pars opercularis	
Pars triangularis	
Pars orbitalis	
Lateral orbitofrontal gyrus	
Medial orbitofrontal gyrus	
Precentral gyrus	
Paracentral lobule	
Frontal pole	
Rostral anterior cingulate	
Caudal anterior cingulate	
**Temporal lobe**	
	ER-alpha and ER-beta distribution in postmortem human brain (Österlund et al., 2000; Osterlund and Hurd, 2001) ER-alpha is downregulated in the hippocampus in postmortem women with AD (Hu et al., 2003; Ishunina et al., 2007), but upregulated across the menopause transition (Ishunina et al., 2007) Reviews of estrogen signaling during the menopause transition (Brinton et al., 2015), in cholinergic (Newhouse and Dumas, 2015), glutamatergic, GABA-ergic, dopaminergic, and serotonergic pathways (Barth et al., 2015)
Entorhinal cortex	Thinner in women who underwent bilateral salpingo-oophorectomy (Zeydan et al., 2019)
Parahippocampal gyrus	Positive association between E2 and volume across menstrual cycle (Zsido et al., 2023) Thinner in women who underwent bilateral salpingo-oophorectomy (Zeydan et al., 2019)
Fusiform gyrus	
Superior temporal gyrus	
Middle temporal gyrus	
Inferior temporal gyrus	
Transverse temporal gyrus	
Temporal pole	

#### 2.3.2 Data analysis plan

Independent-samples *t*-tests and chi-square analyses were conducted to examine demographic differences between *APOE4* carriers and non-carriers. After preliminary data visualization, E2, E1, and p-tau 181 were log-transformed prior to analyses. Multiple linear regressions were used to examine the separate and interactive effects of estrogens (i.e., E2 and E1), AD biomarkers (i.e., Aβ42/40 and Aβ42/p-tau 181), and *APOE4* carrier status on regional brain volumes controlling for age, years of education, race, and BMI. All statistical analyses were conducted using R Statistical Software (Version 4.3.1; R Core Team, 2023).

##### 2.3.2.1 Full sample analyses

We first conducted multiple linear regressions to evaluate the independent associations of E1, E2, and *APOE4* with ROIs. We next conducted multiple linear regressions to evaluate the interactions between: estrogens and *APOE4* status; estrogens and Aβ-42/40 ratio; and estrogens and Aβ-42/p-tau 181 ratio. We controlled for age, years of education, race, and BMI in all analyses.

##### 2.3.2.2 *APOE4* stratified analyses

To determine whether any significant interactive associations of estrogens and AD biomarkers with brain volumes varied by *APOE4* carrier status, we conducted follow-up linear regressions stratifying by *APOE4* carrier status. Specifically, for each ROI volume that was predicted by the interaction of estrogens and AD biomarkers in the full sample, we conducted the same linear regressions as delineated above in the *APOE4*+ and *APOE4*− groups separately again controlling for age, years of education, race, and BMI.

## 3 Results

### 3.1 Participants

Table 2 shows the demographic, clinical, and cognitive characteristics of the 171 participants. The sample included late midlife women (mean age = 59.4 years, age range 45–67 years, mean MoCA = 26.9; race = 83.2% white), all of whom all were postmenopausal (mean self-reported time since final menstrual period = 9.24 years, *SD* = 4.99). Welsch’s *t*-tests revealed that age (*t*(68.3) = −0.567, *p* = 0.57), years of education (*t*(59.6) = 0.537, *p* = 0.59), MoCA score (*t*(62.9) = 0.498, *p* = 0.62), E2 (*t*(62.6) = −0.476, *p* = 0.63), E1 (*t*(75.5) = −1.15, *p* = 0.25), and BMI (*t*(73.1) = 0.692, *p* = 0.49) did not significantly differ by *APOE4* carrier status. A chi-squared analysis showed that racial/ethnic representation did not significantly differ by *APOE4* carrier status, *Χ^2^*(3) = 2.06, *p* = 0.56.

**Table 2:** Participant demographics.

	Full sample (*N* = 171)	*APOE4*− (*N* = 131)	*APOE4*+ (*N* = 40)
Age (yrs)^1^	59.4 (4.03)	59.3 (4.10)	59.7 (3.85)
Race/Ethnicity^2^			
White	141 (82.5%)	109 (83.2%)	32 (80.0%)
Black	23 (13.5%)	16 (12.2%)	7 (17.5%)
Asian/Pacific Islander	3 (1.8%)	2 (1.5%)	1 (2.5%)
Mixed Race	4 (2.3%)	4 (3.1%)	0 (0%)
Education (yrs)^1^	15.8 (2.26)	15.8 (2.21)	15.6 (2.45)
MoCA score^1^	26.9 (2.61)	27.0 (2.62)	26.7 (2.60)
Estradiol (pg/mL)^1^	4.56 (4.91)	4.40 (4.33)	5.08 (6.50)
Estrone (pg/mL)^1^	33.6 (17.0)	33.2 (17.7)	34.9 (14.3)
Body Mass Index^1^	28.3 (6.06)	28.5 (6.25)	27.8 (5.45)

1 Mean (SD).

2 *N*(%).

### 3.2 Regional brain volumes

#### 3.2.1 Full sample

The regression analyses revealed no main effect of *APOE4* carrier status on regional brain volumes (Table 3), E2 levels (*β* = 0.042, *SE* = 0.054, *p* = 0.44), E1 levels (*β* = 0.046, *SE* = 0.035, *p* = 0.19), Aβ-42/40 ratio (*β* = −0.004, *SE* = 0.003, *p* = 0.13), or Aβ-42/p-tau 181 ratio (*β* = −0.046, *SE* = 0.069, *p* = 0.51). E2 levels were associated with greater left caudal middle frontal volume (*p* < 0.05), but were not associated with volumes of any other ROIs (Table 3). E1 was also positively associated with the left caudal middle frontal gyrus volume (*p* = 0.04) and left fusiform volume (*p* < 0.05) only (Table 3). There was no significant interactive association of *APOE4* carrier status and estrogens (E1 or E2) with regional brain volumes (Table 3).

**Table 3:** Main effects of *APOE4* carrier status, E2, E1, and their interactive effects on regional brain volumes.

Region	Hemisphere	*APOE4* Main effect	E2 Main effect	E2 *APOE4	E1 Main effect	E1 * *APOE4*
**Frontal lobe**						
Caudal anterior cingulate	Left	−0.005 (0.005)	0.007 (0.007)	0.014 (0.015)	−0.002 (0.011)	0.001 (0.027)
	Right	−0.001 (0.005)	0.008 (0.007)	−0.003 (0.016)	−0.003 (0.011)	−0.009 (0.028)
Caudal middle frontal	Left	−0.007 (0.012)	0.033 (0.017)*	0.033 (0.037)	0.054 (0.026)*	−0.002 (0.065)
	Right	−0.026(0.015)	0.025 (0.022)	0.054 (0.046)	0.031 (0.034)	0.041 (0.081)
Frontal pole	Left	0.001 (0.002)	−0.002 (0.002)	−0.001 (0.005)	0.001 (0.004)	0.010 (0.009)
	Right	0.000 (0.002)	0.004 (0.003)	0.005 (0.007)	−0.001 (0.005)	0.022 (0.013)
Lateral orbitofrontal	Left	0.009 (0.008)	0.013 (0.011)	0.032 (0.024)	0.017 (0.017)	0.033 (0.042)
	Right	0.002 (0.008)	0.005 (0.011)	0.016 (0.024)	0.011 (0.017)	0.041 (0.042)
Medial orbitofrontal	Left	0.001 (0.006)	0.007 (0.009)	0.022 (0.020)	0.010 (0.014)	0.055 (0.035)
	Right	0.004 (0.007)	0.011 (0.009)	0.018 (0.020)	0.015 (0.014)	0.029 (0.035)
Paracentral	Left	0.002 (0.005)	0.001 (0.007)	0.013 (0.014)	−0.013 (0.010)	−0.006 (0.024)
	Right	0.003 (0.006)	−0.004 (0.008)	0.011 (0.018)	−0.010 (0.013)	0.003 (0.032)
Pars opercularis	Left	0.007 (0.009)	0.002 (0.013)	0.018 (0.027)	0.020 (0.020)	0.041 (0.048)
	Right	−0.002 (0.007)	0.004 (0.010)	0.025 (0.021)	−0.006 (0.015)	0.041 (0.037)
Pars orbitalis	Left	0.004 (0.003)	0.000 (0.004)	0.006 (0.009)	0.003 (0.007)	0.012 (0.016)
	Right	0.003 (0.004)	0.005 (0.006)	0.003 (0.012)	0.011 (0.009)	0.013 (0.021)
Pars triangularis	Left	0.010 (0.007)	0.001 (0.010)	0.009 (0.021)	−0.004 (0.015)	0.012 (0.036)
	Right	0.000 (0.008)	−0.004 (0.011)	0.021 (0.023)	−0.011 (0.017)	0.044 (0.041)
Precentral	Left	0.007 (0.017)	0.002 (0.025)	0.085 (0.052)	0.001 (0.038)	0.035 (0.092)
	Right	0.001 (0.017)	0.008 (0.025)	0.023 (0.053)	0.030 (0.038)	−0.006 (0.092)
Rostral anterior cingulate	Left	−0.005 (0.005)	0.005 (0.007)	0.005 (0.015)	0.004 (0.011)	−0.005 (0.026)
	Right	0.003 (0.004)	0.009 (0.006)	0.006 (0.014)	0.019 (0.010)	0.013 (0.024)
Rostral middle frontal	Left	−0.003 (0.020)	0.010 (0.028)	0.062 (0.062)	0.040 (0.044)	0.038 (0.107)
	Right	−0.012 (0.022)	0.046 (0.032)	0.027 (0.067)	0.042 (0.051)	0.115 (0.118)
Superior frontal	Left	0.008 (0.027)	0.028 (0.039)	0.085 (0.083)	0.036 (0.060)	0.143 (0.146)
	Right	−0.027 (0.028)	0.002 (0.040)	0.131 (0.087)	−0.063 (0.060)	0.023 (0.156)
**Temporal lobe**						
Entorhinal	Left	−0.003 (0.004)	0.000 (0.006)	−0.002 (0.012)	−0.008 (0.008)	0.029 (0.021)
	Right	−0.005 (0.004)	0.006 (0.006)	−0.003 (0.013)	0.002 (0.009)	−0.001 (0.023)
Fusiform	Left	−0.025 (0.017)	0.009 (0.024)	−0.020 (0.051)	0.072 (0.037)*	0.076 (0.088)
	Right	−0.012 (0.015)	−0.015 (0.021)	−0.031 (0.046)	0.002 (0.033)	0.054 (0.081)
Inferior temporal	Left	−0.006 (0.014)	−0.034 (0.020)	0.000 (0.043)	−0.011 (0.031)	0.006 (0.076)
	Right	−0.008 (0.013)	−0.003 (0.018)	−0.008 (0.039)	−0.005 (0.028)	−0.103 (0.068)
Middle temporal	Left	−0.010 (0.013)	−0.001 (0.019)	0.066 (0.041)	0.013 (0.029)	0.062 (0.072)
	Right	0.007 (0.015)	0.011 (0.021)	0.086 (0.045)	0.020 (0.033)	0.125 (0.079)
Parahippocampal	Left	−0.003 (0.004)	0.001 (0.006)	0.002 (0.012)	−0.001 (0.009)	0.013 (0.022)
	Right	−0.001 (0.004)	−0.004 (0.005)	0.001 (0.011)	0.003 (0.008)	0.002 (0.019)
Superior temporal	Left	−0.001 (0.015)	0.035 (0.021)	−0.001 (0.045)	0.039 (0.033)	0.052 (0.080)
	Right	0.015 (0.014)	0.028 (0.020)	0.006 (0.042)	0.027 (0.031)	0.041 (0.075)
Temporal pole	Left	0.004 (0.005)	0.004 (0.007)	−0.001 (0.015)	0.018 (0.011)	−0.001 (0.026)
	Right	−0.002 (0.005)	0.003 (0.007)	0.000 (0.015)	0.005 (0.010)	0.030 (0.026)
Transverse temporal	Left	−0.002 (0.002)	−0.001 (0.003)	0.001 (0.007)	−0.005 (0.005)	−0.004 (0.013)
	Right	0.000 (0.002)	0.000 (0.002)	0.002 (0.005)	−0.005 (0.004)	−0.002 (0.009)

Regression coefficient estimate (standard error). *p < 0.05, **p < 0.01, ***p < 0.001. Bolded values indicate significant associations.

**Figure 1:**
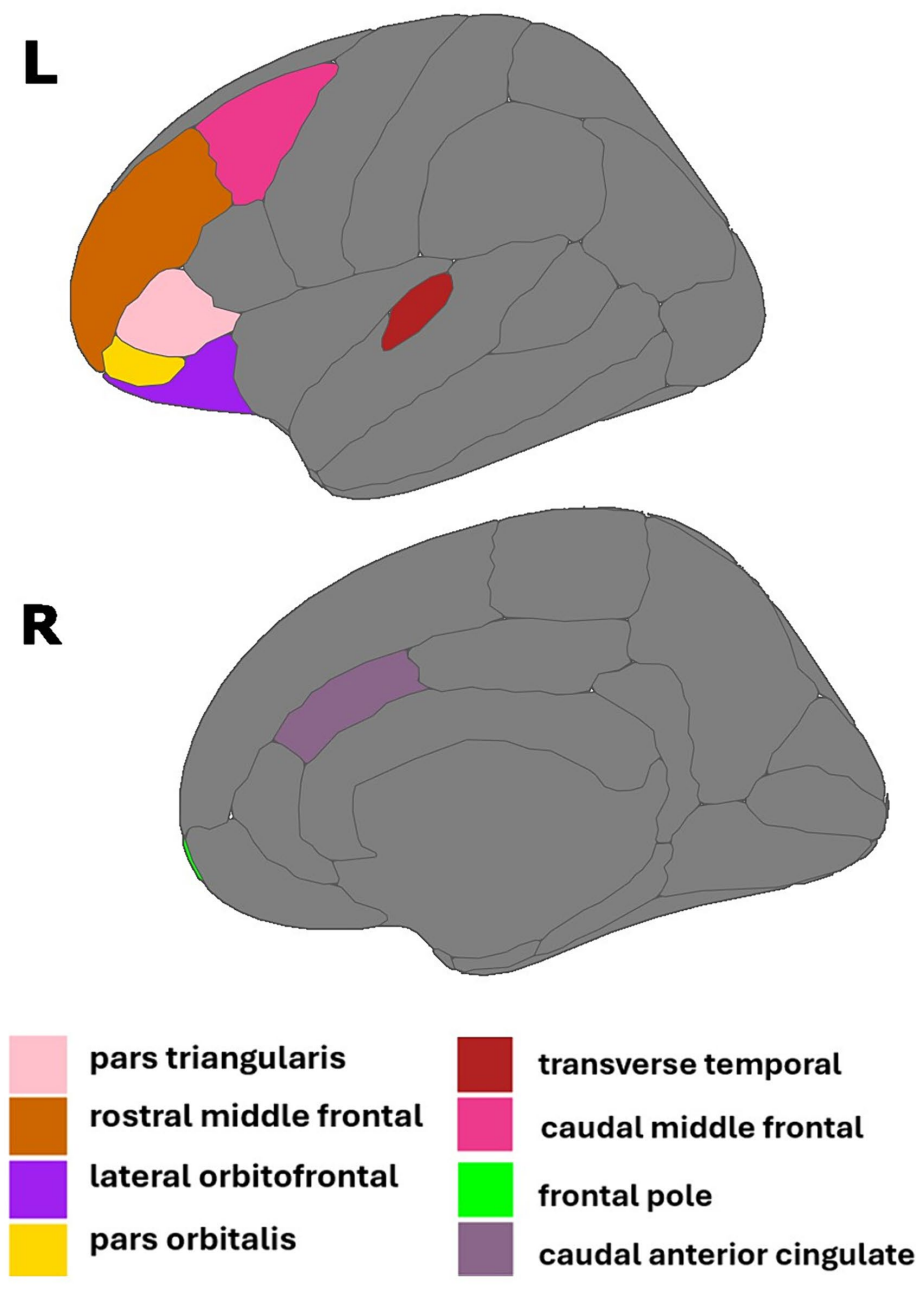
Regions of interest identified in estradiol and AD biomarker analyses. Figure created using the *ggseg* package (Mowinckel and Vidal-Pineiro, 2019) in R Statistical Software (R Core Team, 2023).

**Figure 2:**
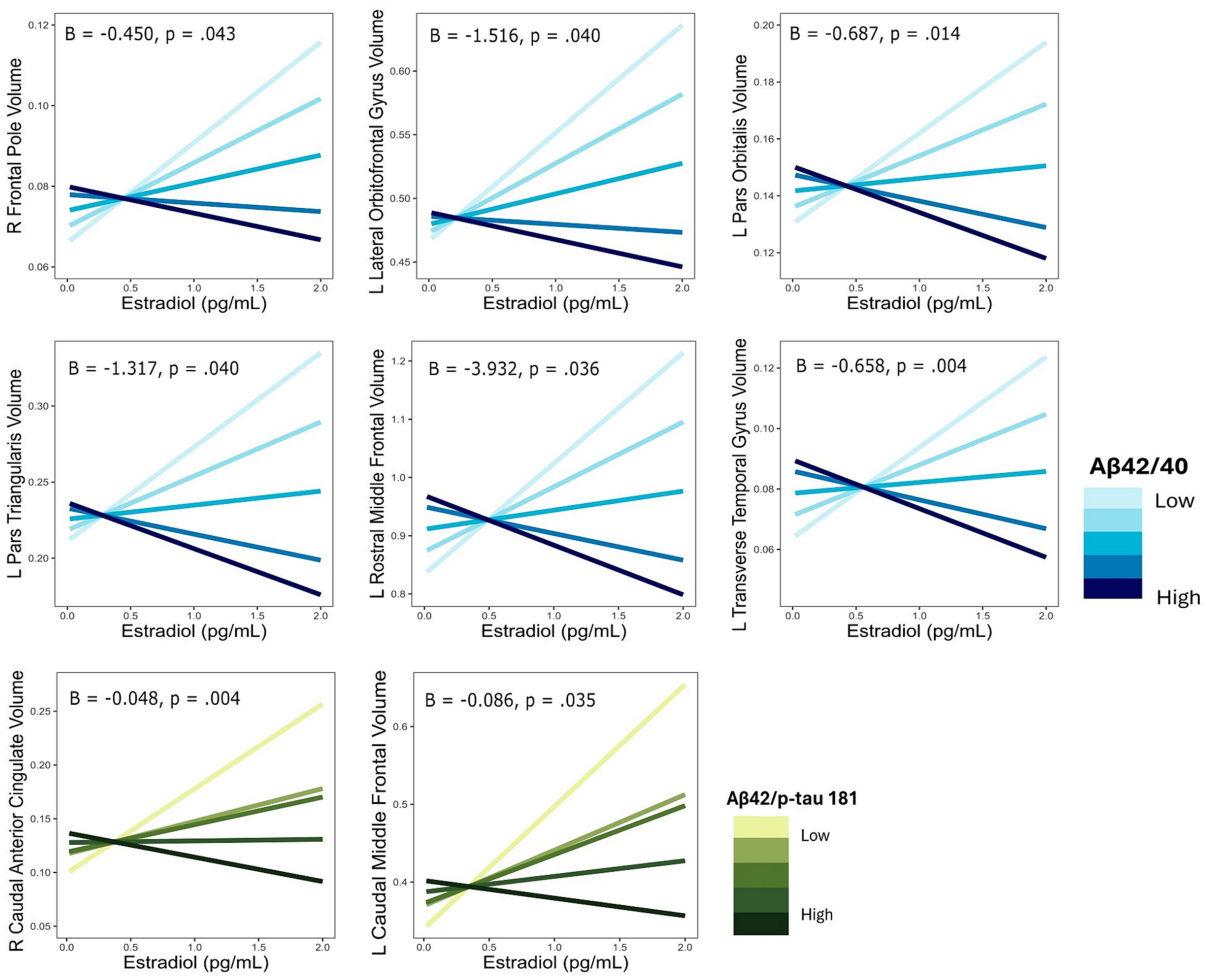
Interactive effects of estradiol and AD biomarker ratios on regional brain volumes. AD biomarker ratios are divided into quintiles. B, regression coefficient estimate for the interaction term; *p*, *p* value for the interaction term.

Multiple linear regressions investigating the interaction of E2 and Aβ-42/40 on regional brain volumes revealed that the association between E2 and the volumes of the right frontal pole (*p* = 0.04), left lateral orbitofrontal gyrus (*p* = 0.04), left pars orbitalis (*p* = 0.01), left pars triangularis (*p* = 0.04), left rostral middle frontal gyrus (*p* = 0.04), and left transverse temporal gyrus (*p* < 0.01) depend on the Aβ-42/40 ratio (Table 4; Figure 1). In addition, regression analyses showed an interactive association of E2 and Aβ-42/p-tau 181 ratio with volumes of the right caudal anterior cingulate gyrus (*p* = <0.01) and the left caudal middle frontal gyrus (*p* = 0.04; Table 4; Figure 1). The pattern of results was similar in all regions, such that the association between E2 levels and brain volume was stronger and more positive for women with lower Aβ42/40 or Aβ42/p-tau 181 ratios, compared to women with higher AD biomarker ratios (Figure 2).

**Table 4:** Interactive effects of estrogens and biomarkers on regional brain volumes in the full sample.

Region	Hemisphere	E2 * Aβ-42/40	E2 * Aβ-42/p-tau 181	E1 * Aβ-42/40	E1 * Aβ-42/p-tau 181
**Frontal lobe**					
Caudal anterior cingulate	Left	0.055 (0.445)	−0.013 (0.017)	0.389 (0.720)	−0.036 (0.027)
	Right	0.157 (0.460)	−0.048 (0.017)**	0.146 (0.745)	−0.072 (0.027)**
Caudal middle frontal	Left	0.199 (1.064)	−0.086 (0.041)*	−0.824 (1.715)	−0.147 (0.064)*
	Right	−0.343 (1.450)	−0.017 (0.053)	−0.935 (2.364)	−0.040 (0.084)
Frontal pole	Left	−0.268 (0.159)	0.004 (0.006)	−0.340 (0.261)	0.015 (0.009)
	Right	−0.450 (0.223)*	0.002 (0.008)	−0.886 (0.363)*	0.020 (0.013)
Lateral orbitofrontal	Left	−1.516 (0.738)*	0.021 (0.028)	−2.619 (1.199)*	0.003 (0.044)
	Right	−0.465 (0.750)	0.015 (0.028)	−1.278 (1.209)	0.015 (0.043)
Medial orbitofrontal	Left	−0.944 (0.612)	−0.013 (0.023)	−0.887 (1.005)	0.043 (0.036)
	Right	−0.575 (0.624)	0.034 (0.023)	−0.972 (1.042)	0.069 (0.036)
Paracentral	Left	−0.151 (0.434)	0.012 (0.016)	0.774 (0.713)	0.027 (0.025)
	Right	0.322 (0.538)	−0.011 (0.020)	1.461 (0.860)	−0.029 (0.032)
Pars opercularis	Left	−0.916 (0.844)	−0.028 (0.031)	−2.837 (1.353)*	−0.039 (0.049)
	Right	−1.017 (0.664)	0.005 (0.025)	−1.746 (1.066)	−0.030 (0.039)
Pars orbitalis	Left	−0.687 (0.280)*	0.015 (0.010)	−0.917 (0.453)*	0.015 (0.016)
	Right	−0.307 (0.373)	−0.003 (0.013)	−0.504 (0.610)	−0.003 (0.021)
Pars triangularis	Left	−1.317 (0.642)*	−0.009 (0.024)	−2.633 (1.037)*	−0.036 (0.038)
	Right	−0.696 (0.735)	0.017 (0.026)	−0.651 (1.306)	0.005 (0.041)
Precentral	Left	−1.771 (1.632)	0.028 (0.060)	−0.794 (2.666)	0.079 (0.095)
	Right	−2.847 (1.632)	0.056 (0.061)	−4.565 (2.704)	0.035 (0.095)
Rostral anterior cingulate	Left	0.427 (0.446)	0.014 (0.017)	0.393 (0.725)	−0.034 (0.027)
	Right	0.634 (0.398)	−0.025 (0.015)	0.585 (0.640)	−0.047 (0.024)*
Rostral middle frontal	Left	−3.932 (1.873)*	−0.098 (0.070)	−4.946 (3.064)	−0.102 (0.111)
	Right	−0.001 (2.125)	0.038 (0.076)	−1.112 (3.806)	0.026 (0.121)
Superior frontal	Left	−2.020 (2.579)	−0.041 (0.096)	−7.127 (4.156)	−0.093 (0.150)
	Right	−0.527 (2.610)	−0.003 (0.094)	1.283 (4.241)	0.020 (0.149)
**Temporal lobe**					
Entorhinal	Left	0.064 (0.372)	0.002 (0.014)	0.595 (0.601)	0.006 (0.022)
	Right	0.040 (0.388)	−0.011 (0.015)	0.063 (0.634)	0.024 (0.023)
Fusiform	Left	0.163 (1.607)	0.033 (0.058)	−1.447 (2.587)	0.045 (0.091)
	Right	−0.031 (1.428)	0.060 (0.052)	1.088 (2.333)	0.074 (0.083)
Inferior temporal	Left	2.412 (1.328)	−0.020 (0.049)	2.827 (2.245)	0.002 (0.078)
	Right	1.617 (1.217)	−0.009 (0.045)	1.504 (2.046)	0.027 (0.071)
Middle temporal	Left	−0.284 (1.287)	−0.026 (0.047)	−2.101 (2.079)	0.034 (0.074)
	Right	1.330 (1.418)	−0.045 (0.051)	−0.614 (2.319)	−0.069 (0.081)
Parahippocampal	Left	−0.596 (0.375)	−0.004 (0.014)	−0.534 (0.615)	−0.002 (0.022)
	Right	−0.524 (0.337)	0.004 (0.013)	−0.524 (0.549)	0.024 (0.020)
Superior temporal	Left	−1.764 (1.396)	0.031 (0.051)	−3.983 (2.262)	−0.003 (0.080)
	Right	−1.509 (1.314)	0.016 (0.047)	−2.222 (2.156)	−0.006 (0.076)
Temporal pole	Left	−0.717 (0.454)	−0.004 (0.017)	−0.971 (0.737)	0.021 (0.027)
	Right	−0.708 (0.449)	0.012 (0.016)	−1.025 (0.733)	0.045 (0.026)
Transverse temporal	Left	−0.658 (0.226)**	0.014 (0.008)	−1.277 (0.363)***	0.002 (0.014)
	Right	−0.207 (0.157)	0.010 (0.006)	−0.348 (0.255)	0.006 (0.009)

Regression coefficient estimate (standard error). *p < 0.05, **p < 0.01, ***p < 0.001. Bolded values indicate significant associations.

**Figure 3:**
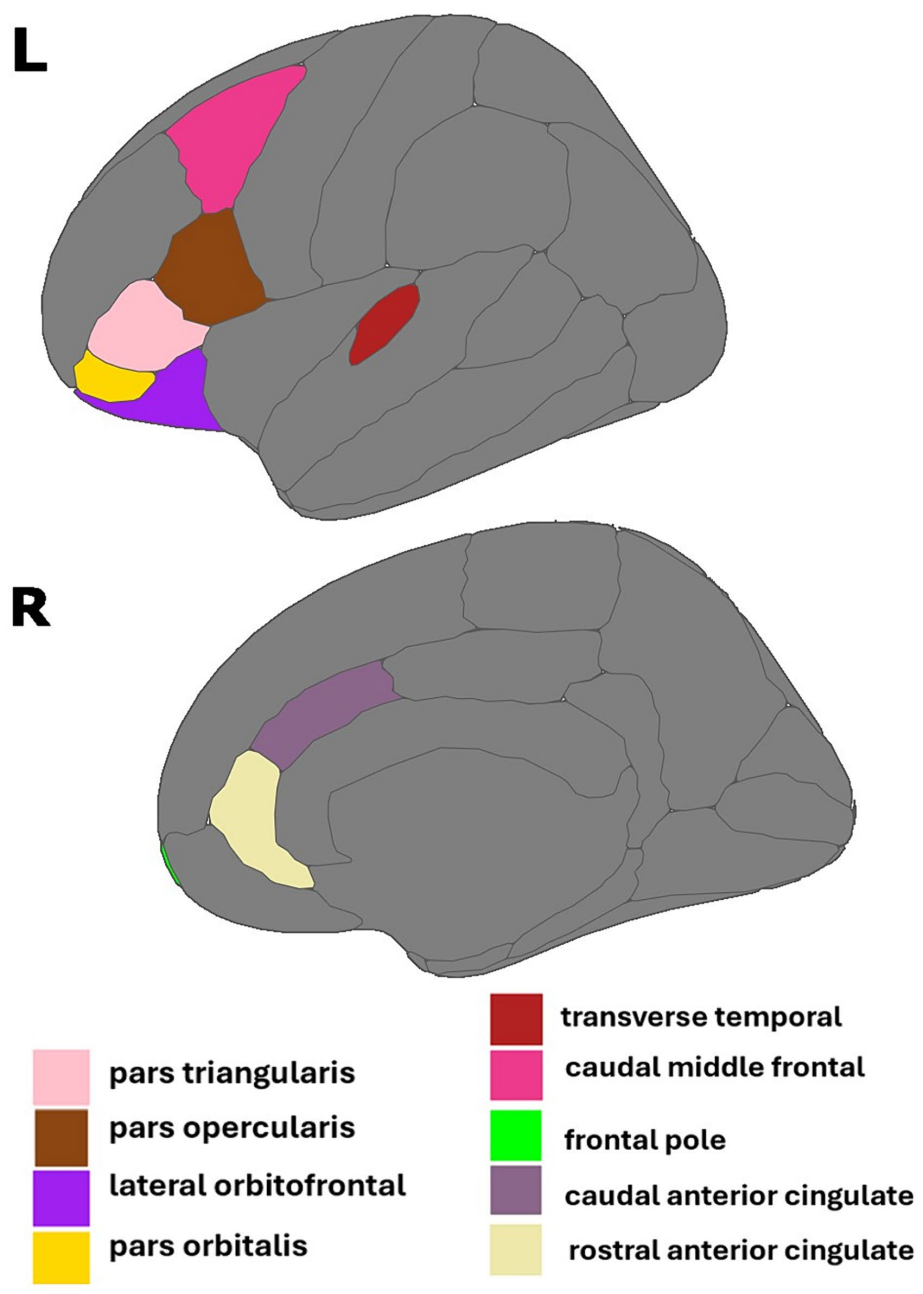
Regions of interest identified in estrone and AD biomarker analyses. Figure created using the *ggseg* package (Mowinckel and Vidal-Pineiro, 2019) in R Statistical Software (R Core Team, 2023).

**Figure 4:**
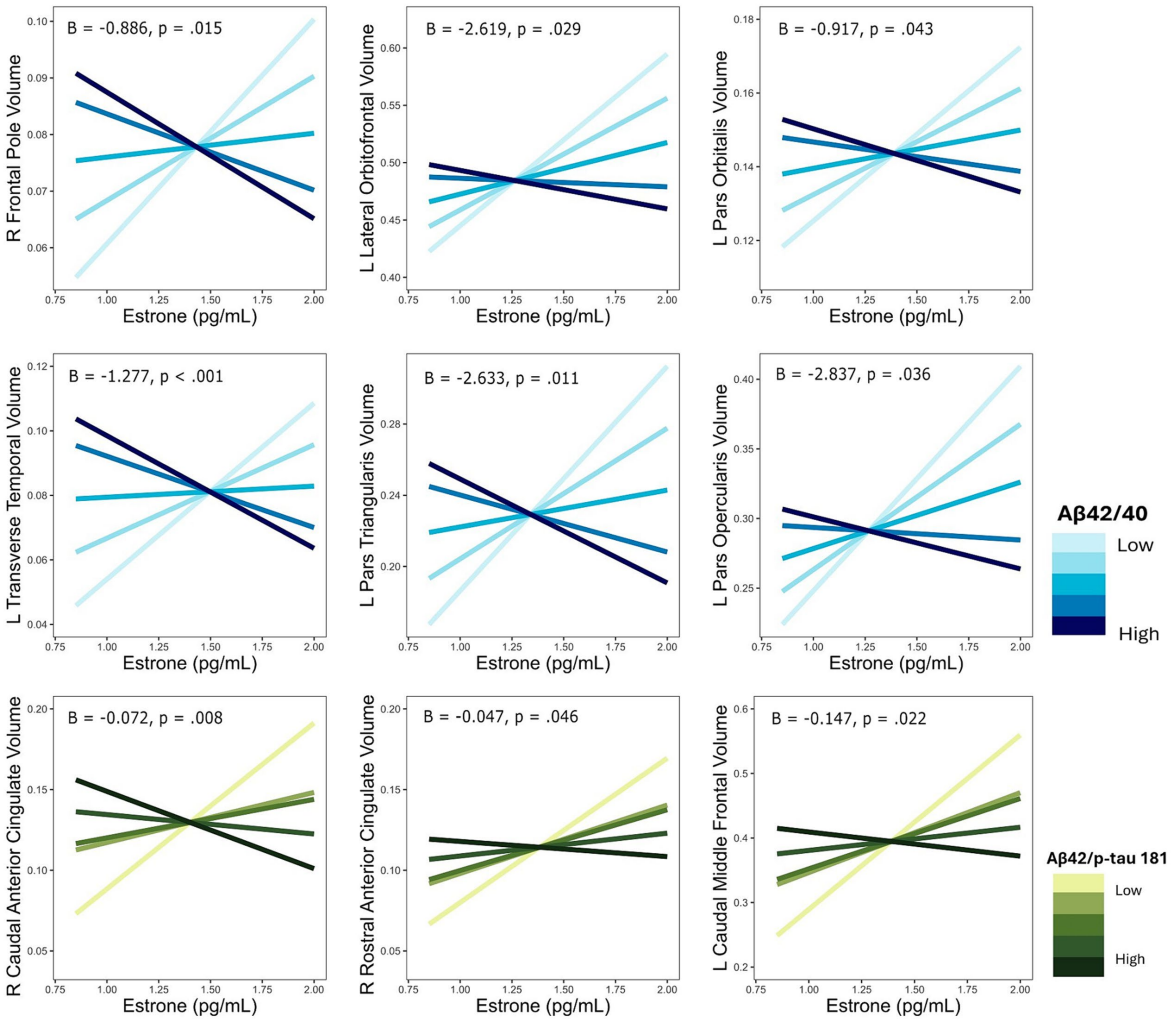
Interactive effects of estrone and AD biomarker ratios on regional brain volumes. AD biomarker ratios are divided into quintiles. B, regression coefficient estimate for the interaction term; *p, p* value for the interaction term.

The interaction of E1 and Aβ-42/40 was associated with volumes of the right frontal pole (*p* = 0.01), left lateral orbitofrontal gyrus (*p* = 0.03), left pars orbitalis (*p* = 0.04), left pars triangularis (*p* = 0.01), left pars opercularis (*p* = 0.04), and left transverse temporal gyrus (*p* < 0.001; Table 4; Figure 3). Similarly, there was an interaction of E1 and Aβ-42/p-tau 181 ratio on the volumes of the right caudal anterior cingulate gyrus (*p* < 0.01), right rostral anterior cingulate gyrus (*p* = 0.046), and left caudal middle frontal gyrus (*p* = 0.02; Table 4; Figure 3). The pattern of results is similar among all brain regions, with greater brain volumes as E1 increases and AD biomarker ratios decrease (Figure 4).

#### 3.2.2 *APOE4* stratified

**Figure 5:**
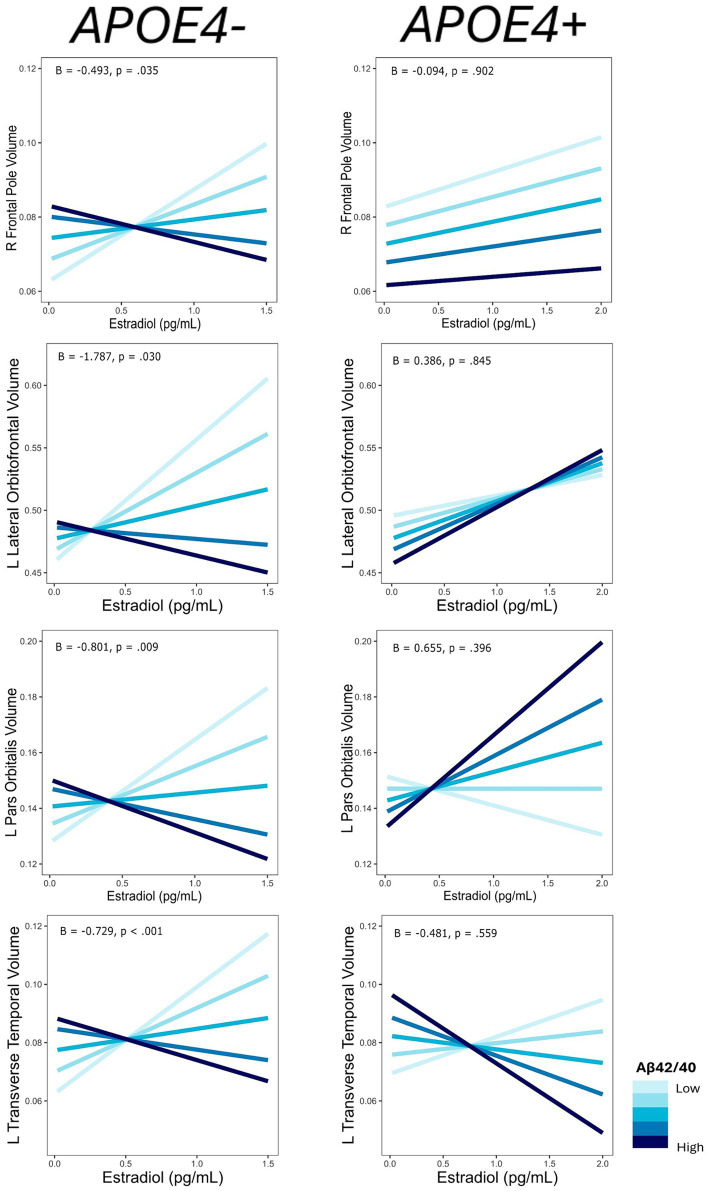
Interactive effects of estradiol and Aβ42/40 on regional brain volumes, stratified by *APOE4* carrier status. Aβ42/40 is divided into quintiles. B, regression coefficient estimate for the interaction term; *p*, *p* value for the interaction term.

**Figure 6:**
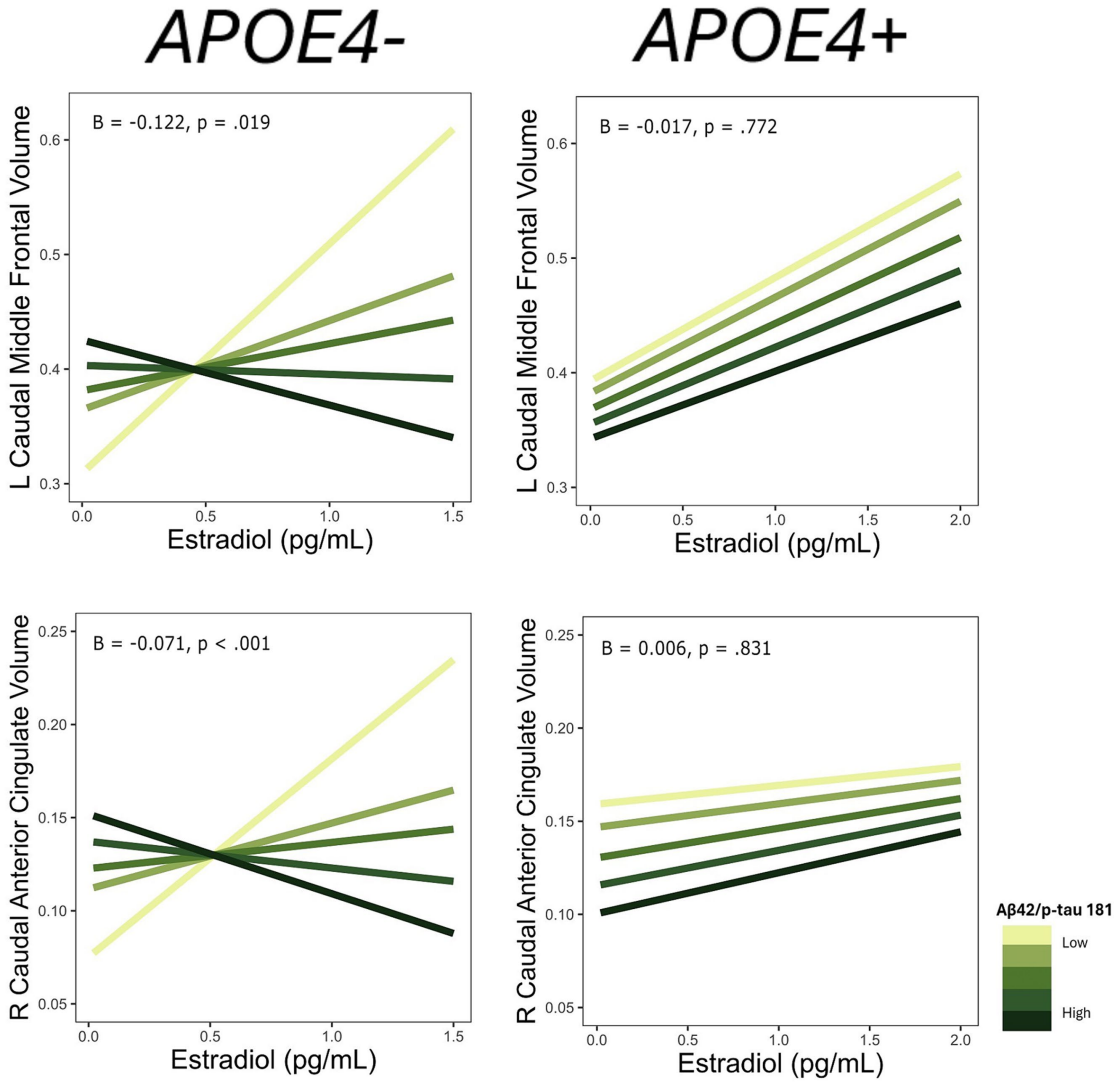
Interactive effects of estradiol and Aβ42/p-tau 181 on regional brain volumes, stratified by *APOE4* carrier status. Aβ42/p-tau 181 is divided into quintiles. B, regression coefficient estimate for the interaction term; *p*, *p* value for the interaction term.

For each ROI where an interaction of E2 and AD biomarkers was observed, we conducted the same regression analyses stratified by *APOE4* and non-*APOE4* carriers. There was an interactive association of E2 and Aβ-42/40 ratio on the volumes of the right frontal pole (*p* = 0.04), left lateral orbitofrontal gyrus (*p* = 0.03), left pars orbitalis (*p* < 0.01), and left transverse temporal gyrus (*p* < 0.001) among the non-*APOE4* carrier group only (Table 5). Additionally, there was an interaction of E2 and Aβ-42/p-tau 181 ratio on volumes of the right caudal anterior cingulate gyrus (*p* < 0.001) and left caudal middle frontal gyrus (*p* = 0.02), among the non-*APOE4* carrier group only (Table 5). When comparing women with lower (more severe) AD biomarker ratios, *APOE4*− women showed a stronger positive relationship between E2 and brain volumes in all ROIs compared to *APOE4*+ women (Figures 5, 6). Among women with higher (normal) AD biomarker ratios, non-*APOE4* carriers show a weak negative relationship between E2 and brain volumes in all significant ROIs. In *APOE4* carriers, the pattern of results differs by brain region, yet there were no interactions of E2 and AD biomarkers on brain volume and thus the biomarker slopes are not significantly different from each other (Figures 5, 6).

**Table 5:** Interactive effects of estrogens and biomarkers on regional brain volumes, stratified by *APOE4* genotype.

Region	*APOE4*−	*APOE4*+
	E2 * Aβ-42/40	
Right frontal pole	−0.493 (0.234)*	−0.094 (0.763)
Left lateral orbitofrontal	−1.787 (0.823)*	0.386 (1.977)
Left pars orbitalis	−0.801 (0.309)**	0.655 (0.772)
Left pars triangularis	−1.022 (0.695)	−1.750 (2.018)
Left rostral middle frontal	−3.556 (2.023)	−6.109 (6.241)
Left transverse temporal	−0.729 (0.220)***	−0.481 (0.825)
	E2 * Aβ-42/pTau-181	
Right caudal anterior cingulate	−0.071 (0.020)***	0.006 (0.029)
Left caudal middle frontal	−0.122 (0.052)*	−0.017 (0.057)
	E1 * Aβ-42/40	
Right frontal pole	−0.725 (0.377)	−0.852 (1.235)
Left lateral orbitofrontal	−2.756 (1.331)*	−0.502 (3.417)
Left pars opercularis	−1.720 (1.415)	−7.367 (4.554)
Left pars orbitalis	−1.016 (0.502)*	0.371 (1.254)
Left pars triangularis	−1.941 (1.120)	−5.270 (3.312)
Left transverse temporal	−1.360 (0.351)***	−0.942 (1.364)
	E1 * Aβ-42/pTau-181	
Right caudal anterior cingulate	−0.092 (0.031)**	−0.016 (0.057)
Right rostral anterior cingulate	−0.055 (0.028)	−0.050 (0.041)
Left caudal middle frontal	−0.168 (0.077)*	−0.050 (0.116)

Regression coefficient estimate (standard error). *p < 0.05, **p < 0.01, ***p < 0.001. Bolded values indicate significant associations.

**Figure 7:**
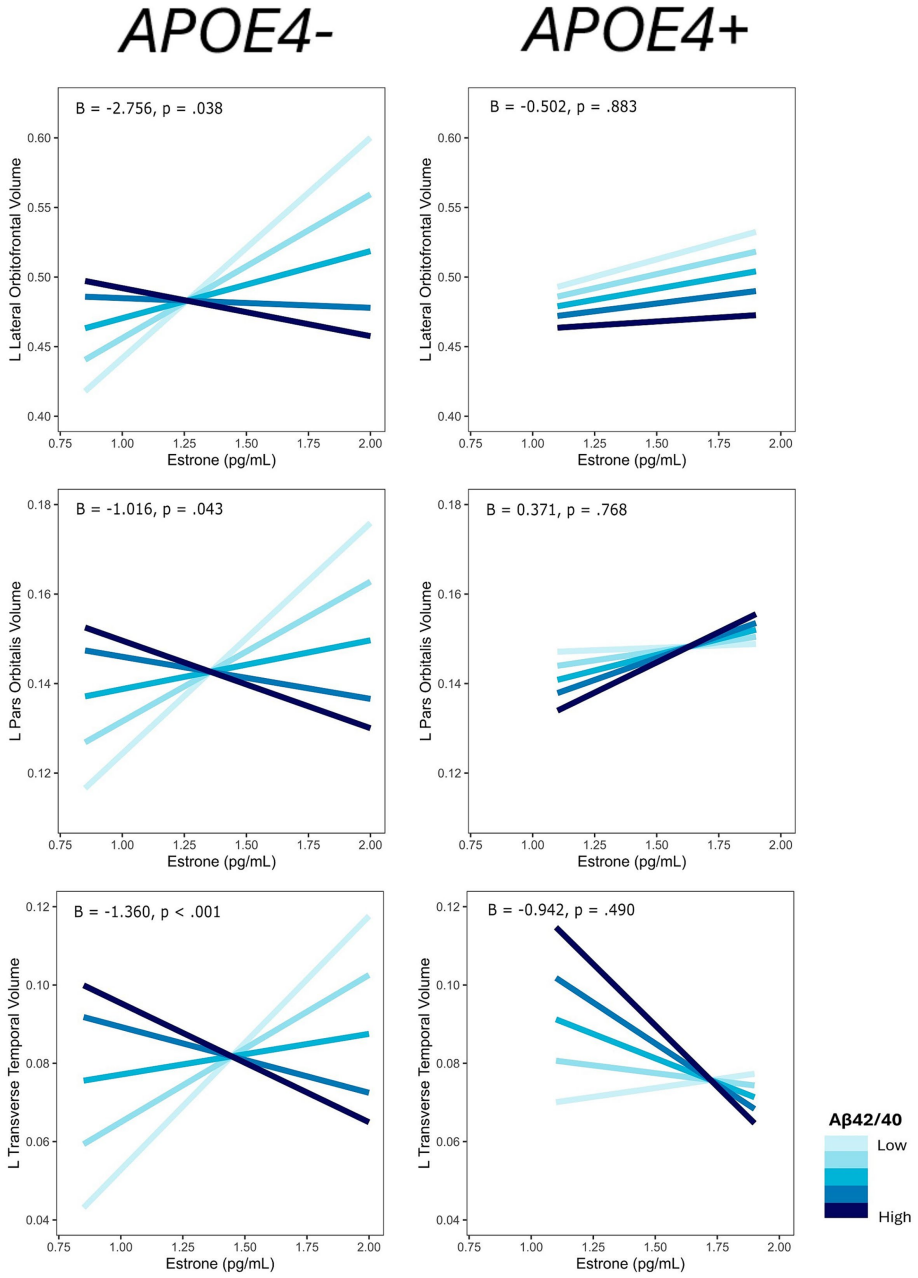
Interactive effects of estrone and Aβ42/40 on regional brain volumes, stratified by *APOE4* carrier status. Aβ42/40 is divided into quintiles. B, regression coefficient estimate for the interaction term; *p*, *p* value for the interaction term.

**Figure 8:**
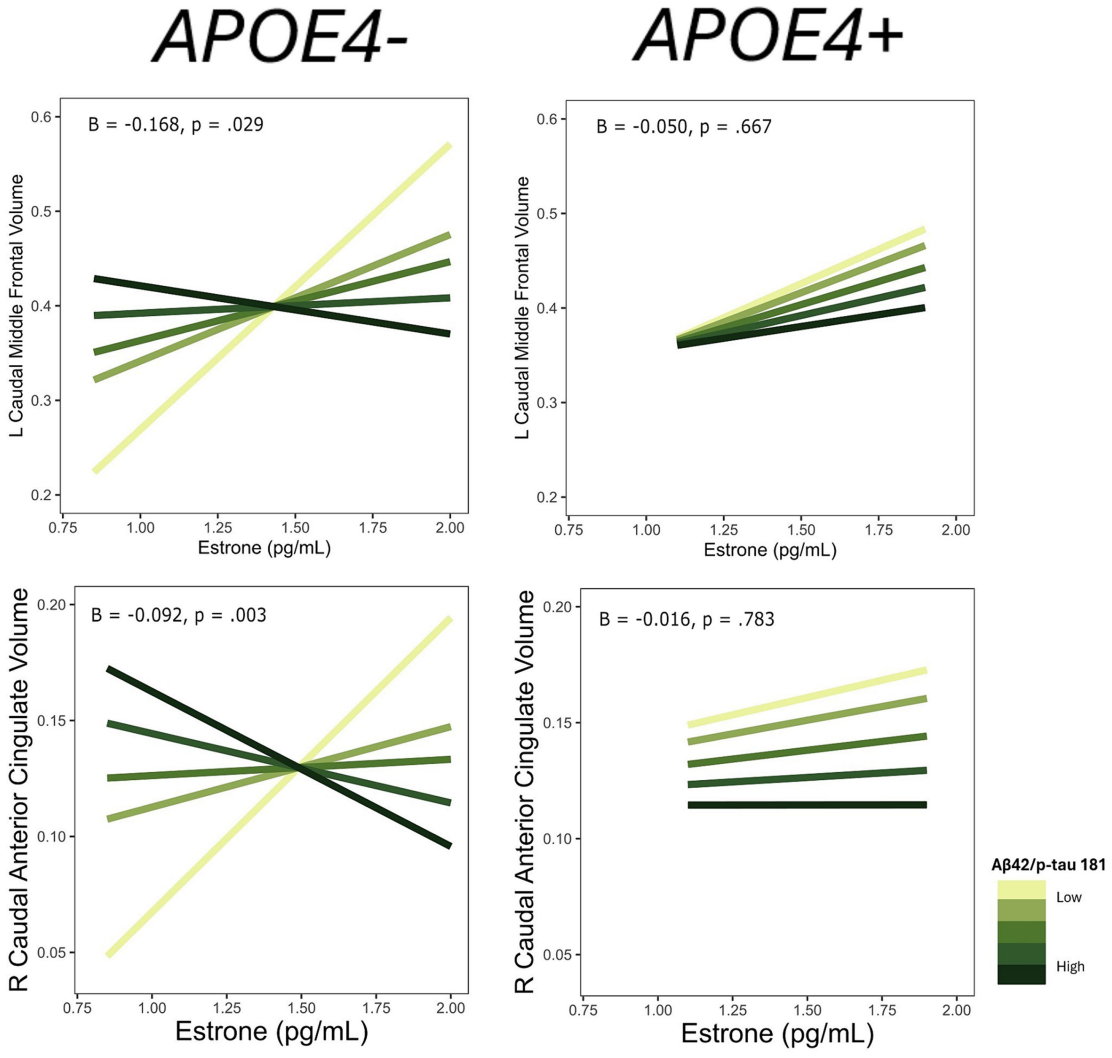
Interactive effects of estrone and Aβ42/p-tau 181 on regional brain volumes, stratified by *APOE4* carrier status. Aβ42/p-tau 181 is divided into quintiles. B, regression coefficient estimate for the interaction term; *p*, *p* value for the interaction term.

Among the ROIs associated with the interaction of E1 and AD biomarkers above, we conducted the same regression analyses stratified by *APOE4* carrier status. Of those ROIs, there was an interaction of E1 and Aβ-42/40 ratio on the volumes of the left lateral orbitofrontal gyrus (*p* = 0.04), left pars orbitalis (*p* = 0.04), and left transverse temporal gyrus (*p* < 0.001) among non-*APOE4* carriers only (Table 5). There was also an interaction of E1 and Aβ-42/p-tau 181 ratio on volumes of the right caudal anterior cingulate gyrus (*p* < 0.01) and left caudal middle frontal gyrus (*p* = 0.03) among non-*APOE4* carriers only (Table 5). When comparing women with lower (more severe) AD biomarker ratios by *APOE4* groups, *APOE4*− women have a stronger positive relationship between E1 and brain volumes compared to *APOE4*+ women (Figures 7, 8). Among women with higher (normal) AD biomarker ratios, *APOE*− women showed a weak negative relationship between E1 and brain volumes in all ROIs. There were no significant interactions of E1 and either AD biomarker on brain volume among *APOE4* carriers (Figures 7, 8).

## 4 Discussion

In a sample of late midlife, postmenopausal women, we investigated the interactive associations of endogenous estrogens and plasma AD biomarkers with volumes in brain areas rich in estrogen receptors. We had a particular interest in whether these associations differed by *APOE4* carrier status. Independently, endogenous estrogens and *APOE4* carrier status were not significantly associated with brain volumes, nor were there interactions between estrogen levels and *APOE4* carrier status in relation to regional brain volumes. However, there were interactions between estrogens and plasma AD biomarkers on volume of several brain regions; both E1 and E2 had a stronger positive association with regional brain volumes among women with worse AD biomarkers as measured by lower Aβ42/40 or Aβ42/p-tau 181 ratios. Furthermore, *APOE4-*stratified analyses revealed that these interactions were primarily driven by non-*APOE4* carriers. Among *APOE4* carriers, endogenous estrogens do not associate with the volume of these brain regions regardless of the severity of AD biomarkers. Overall, these findings suggest that both endogenous E1 and E2 may positively influence brain volume in late reproductive women with higher risk of AD as evidenced by AD biomarkers but not as evidenced by *APOE4* carrier status.

In analyses focusing on estrogens and *APOE4* carrier status, we found no evidence that the effect of endogenous estrogens on brain volume varied with *APOE4* status. The effects of estrogens on women’s brain structure in the postmenopause have primarily been evaluated in randomized controlled trials of MHT, which produces higher levels of estrogen (Resnick et al., 2009; Kantarci et al., 2016b, 2018). The findings across those studies are inconsistent. The Women’s Health Initiative Memory Study (WHIMS) found that women randomized to receive conjugated equine estrogens (CEE) had smaller frontal lobe volumes and slightly lower hippocampal volumes compared to women on placebo (Resnick et al., 2009). In the Kronos Early Estrogen Prevention Study (KEEPS), a randomized trial of MHT in early postmenopausal women, there was no effect of MHT on whole brain volumes after two years of use of transdermal E2 or CEE, but women on CEE had higher rates of ventricular expansion compared to placebo (Kantarci et al., 2016b). After three years of MHT use in the KEEPS, women randomized to receive transdermal E2 showed lower longitudinal decreases in dorsolateral prefrontal cortex (dlPFC) volume compared to placebo (Kantarci et al., 2018). E2 treatment also increased dlPFC volume in non-human primates (Hao et al., 2006) and enhanced functional connectivity between the dlPFC and hippocampus in postmenopausal women (Ottowitz et al., 2008). In the observational study of women in the European Prevention of Alzheimer’s Disease (EPAD) cohort, MHT was associated with larger entorhinal and amygdala volume in *APOE4* carriers (Saleh et al., 2023). *APOE4* carriers in EPAD also had larger hippocampal volumes with earlier age at MHT initiation (Saleh et al., 2023). This is consistent with findings from a large sample of MHT users (*n* = 5,164) from the UK Biobank that earlier MHT initiation is associated with less evident brain aging (calculated with measures of cortical thickness, cortical and subcortical volumes) among *APOE4*+ women only (de Lange et al., 2020). Furthermore, in a small sample (*n* = 25) of *APOE4*+ postmenopausal women in Beijing, China, women taking MHT for at least four years (*n* = 14) had greater hippocampal volumes than women who had never used MHT (*n* = 11; Yue et al., 2007). Here, we found that higher levels of endogenous E2 and E1 were associated with greater volume of the left caudal middle frontal gyrus, an area within the dlPFC. However, our findings generally indicate that endogenous estrogens in the postmenopause do not have a strong direct effect on regional brain volumes. Rather, our results suggest that the effect of endogenous estrogens on brain structure may depend on AD biomarker level and *APOE4* carrier status, with higher endogenous estrogen levels being most advantageous for non-*APOE4* carriers with lower levels of AD biomarkers. While the low levels of AD biomarkers captured in our sample do not meet the cut-offs to predict cognitive decline or dementia (Blennow et al., 2019; Brum et al., 2022), our findings indicate that biomarker levels within this range may affect brain structure when considering additional contributing factors such as estrogens and *APOE* genotype.

In our full sample analyses, we identified ten regions wherein the relationship between estrogens and volume depended on blood-based AD biomarkers. The regions and patterns of associations were similar for E1 and E2 which suggests that the effects are not specific to a particular estrogen type. The ten regions with an interaction of estrogens and AD biomarkers were primarily located in the frontal lobe, several of which were within the ventral and dorsolateral PFC (e.g., pars orbitalis, pars triangularis, pars opercularis, caudal middle frontal gyrus). The PFC is a key region for executive function, working memory and attentional control (Fuster, 2001), and connectivity between the PFC and the hippocampus is crucial for episodic memory performance (Nyberg, 2016; Eichenbaum, 2017). Cognitive abilities mediated by the PFC, including working memory, encoding-related strategic processing, and executive functions, are maintained by estradiol, as evidenced by studies of oophorectomy among women (Phillips and Sherwin, 1992), pharmacological suppression of ovarian hormones via gonadotropin releasing hormone agonists (Craig et al., 2008), and estrogen supplementation in non-human primates (Rapp et al., 2003). Thus, the findings here may have implications for women’s performance in these cognitive domains.

Although to our knowledge this is the first study to examine interactive associations of endogenous estrogens and amyloid with brain volume, other work examined interactions between biological sex and amyloid on brain volume and cognitive functions. For example, there is evidence of a sex difference in the association of Aβ-42 with cognitive function and brain volume, with females showing stronger associations of Aβ-42 with left hippocampal atrophy and with declining memory and executive function performance (Koran et al., 2017). Similar sex differences are observed in a triple transgenic animal model of AD, where females show higher Aβ accumulation in the frontal cortex and more severe cognitive deficits compared to males, an effect driven by prenatal exposure to estrogen in the females (Carroll et al., 2010). Circulating estrogens also appear to contribute to the sex difference in that transgenic mouse model, as depletion of sex steroid hormones via oophorectomy increased Aβ accumulation and decreased memory performance while administration of estradiol, but not progesterone, prevented these effects (Carroll et al., 2007). Together, these studies raise the possibility that endogenous estrogen may drive some of the sex differences in brain health.

Our findings demonstrate that postmenopausal women with the most severe AD biomarkers (i.e., low ratios of Aβ42/40 or Aβ42/p-tau 181) benefit more from higher levels of endogenous estrogens, compared to women with less severe AD biomarker ratios. That association, however, varied by *APOE4* carrier status with benefit among non-*APOE4* carriers but not among *APOE4* carriers. These results were not driven by different levels of AD biomarkers between the two groups, as AD biomarkers did not differ by *APOE4* carrier status in this group of postmenopausal women. Overall, these data suggest that regardless of AD biomarker levels, late midlife women who carry the *APOE4* allele may be insensitive to the effects of high-normal levels of endogenous estrogen on cortical regions that support memory performance. Given prior work (Kantarci et al., 2016a; Saleh et al., 2023), it may be that higher levels of estrogen, such as those found in MHT, are needed to confer benefits in *APOE4* carriers.

The study has notable strengths and limitations. This is the first study in late midlife women to examine whether the effects of estrogens and AD biomarkers on brain volumes differ by *APOE4* carrier status. The study looked not only at E2 but also E1, the predominant estrogen in postmenopausal women. We had a well-characterized sample of midlife women and used highly sensitive estrogen assays. Even with a sample size of 171, we had limited power to test three-way interactive associations (i.e., estrogens by AD biomarkers by *APOE4* carrier status). Our statistical approach therefore examined interactive associations of estrogens and AD biomarkers by *APOE4* carrier status. We did not control for multiple comparisons but *a priori* limited our analyses to 21 ROIs in the temporal and prefrontal cortex, regions with higher densities of estrogen receptors. Some of the findings may be chance findings, and therefore future work is needed to determine if our findings replicate in other samples.

## 5 Conclusion

In conclusion, we demonstrate that late midlife postmenopausal women with the most severe AD biomarkers benefit most from greater endogenous estrogen levels. This finding is driven by *APOE4* non-carriers, indicating that the direct and interactive effects of estrogens may not be beneficial for brain structure in *APOE* ε4 carriers regardless of AD biomarkers severity. These findings suggest that compared to non-carriers, *APOE* ε4+ women may be at increased risk of AD due to the relative insensitivity to potential benefits of endogenous estrogens on brain volumes in the postmenopause.

## Data availability statement

The data analyzed in this study is subject to the following licenses/restrictions: the data and associated materials that support the findings of this study are available from the corresponding author on reasonable request. Requests to access these datasets should be directed to pmaki1@uic.edu.

## Ethics statement

The studies involving humans were approved by University of Pittsburgh Human Research Protection Office. The studies were conducted in accordance with the local legislation and institutional requirements. The participants provided their written informed consent to participate in this study.

## Author contributions

KW: Conceptualization, Formal analysis, Methodology, Visualization, Writing – original draft, Writing – review & editing. RS: Conceptualization, Formal analysis, Methodology, Writing – original draft. RT: Methodology, Writing – review & editing, Funding acquisition, Project administration, Supervision. MW: Methodology, Supervision, Writing – review & editing. HA: Methodology, Writing – review & editing. AC: Writing – review & editing. MK: Methodology, Writing – review & editing. TK: Methodology, Writing – review & editing. CD: Writing – review & editing. PM: Conceptualization, Funding acquisition, Methodology, Project administration, Supervision, Writing – original draft, Writing – review & editing.

## References

[ref1] Alvarez-De-La-Rosa M. Silva I. Nilsen J. Pérez M. M. García-Segura L. M. Ávila J.. (2005). Estradiol prevents neural tau hyperphosphorylation characteristic of Alzheimer’s disease. Ann. N. Y. Acad. Sci. 1052, 210–224. doi: 10.1196/annals.1347.016, PMID: 16024764

[ref2] Alzheimer’s Association (2023). 2023 Alzheimer’s disease facts and figures. Alzheimers Dement. 19, 1598–1695. doi: 10.1002/alz.13016, PMID: 36918389

[ref3] Amtul Z. Wang L. Westaway D. Rozmahel R. F. (2010). Neuroprotective mechanism conferred by 17beta-estradiol on the biochemical basis of Alzheimer’s disease. Neuroscience 169, 781–786. doi: 10.1016/j.neuroscience.2010.05.031 20493928

[ref4] Barth C. Villringer A. Sacher J. (2015). Sex hormones affect neurotransmitters and shape the adult female brain during hormonal transition periods. Front. Neurosci. 9:37. doi: 10.3389/fnins.2015.00037, PMID: 25750611 PMC4335177

[ref5] Blennow K. Shaw L. M. Stomrud E. Mattsson N. Toledo J. B. Buck K.. (2019). Predicting clinical decline and conversion to Alzheimer’s disease or dementia using novel Elecsys Aβ(1–42), pTau and tTau CSF immunoassays. Sci. Rep. 9:19024. doi: 10.1038/s41598-019-54204-z, PMID: 31836810 PMC6911086

[ref7] Brinton R. D. Yao J. Yin F. Mack W. J. Cadenas E. (2015). Perimenopause as a neurological transition state. Nat. Rev. Endocrinol. 11, 393–405. doi: 10.1038/nrendo.2015.82, PMID: 26007613 PMC9934205

[ref8] Brum W. S. De Bastiani M. A. Bieger A. Therriault J. Ferrari-Souza J. P. Benedet A. L.. (2022). A three-range approach enhances the prognostic utility of CSF biomarkers in Alzheimer’s disease. Alzheimers Dement (N Y) 8:e12270. doi: 10.1002/trc2.12270, PMID: 35310530 PMC8918110

[ref9] Carroll J. C. Rosario E. R. Chang L. Stanczyk F. Z. Oddo S. LaFerla F. M.. (2007). Progesterone and estrogen regulate Alzheimer-like neuropathology in female 3xTg-AD mice. J. Neurosci. 27, 13357–13365. doi: 10.1523/JNEUROSCI.2718-07.2007, PMID: 18045930 PMC6673397

[ref10] Carroll J. C. Rosario E. R. Kreimer S. Villamagna A. Gentzschein E. Stanczyk F. Z.. (2010). Sex differences in β-amyloid accumulation in 3xTg-AD mice: role of neonatal sex steroid hormone exposure. Brain Res. 1366, 233–245. doi: 10.1016/j.brainres.2010.10.009, PMID: 20934413 PMC2993873

[ref11] Corder E. H. Ghebremedhin E. Taylor M. G. Thal D. R. Ohm T. G. Braak H. (2004). The biphasic relationship between regional brain senile plaque and neurofibrillary tangle distributions: modification by age, sex, and *APOE* polymorphism. Ann. N. Y. Acad. Sci. 1019, 24–28. doi: 10.1196/annals.1297.005, PMID: 15246987

[ref12] Craig M. C. Fletcher P. C. Daly E. M. Rymer J. Brammer M. Giampietro V.. (2008). Reversibility of the effects of acute ovarian hormone suppression on verbal memory and prefrontal function in pre-menopausal women. Psychoneuroendocrinology 33, 1426–1431. doi: 10.1016/j.psyneuen.2008.08.006, PMID: 18835663

[ref13] Damoiseaux J. S. Seeley W. W. Zhou J. Shirer W. Coppola G. Karydas A.. (2012). Gender modulates the APOE 4 effect in healthy older adults: convergent evidence from functional brain connectivity and spinal fluid tau levels. J. Neurosci. 32, 8254–62. 22699906 10.1523/JNEUROSCI.0305-12.2012 PMC3394933

[ref14] De Lange A. G. Barth C. Kaufmann T. Maximov I. I. Van Der Meer D. Agartz I.. (2020). Women’s brain aging: effects of sex-hormone exposure, pregnancies, and genetic risk for Alzheimer’s disease. Hum. Brain Mapp. 41, 5141–5150. doi: 10.1002/hbm.25180, PMID: 32856754 PMC7670641

[ref15] Depypere H. Vergallo A. Lemercier P. Lista S. Benedet A. Ashton N.. (2023). Menopause hormone therapy significantly alters pathophysiological biomarkers of Alzheimer’s disease. Alzheimers Dement. 19, 1320–1330. doi: 10.1002/alz.12759, PMID: 36218064

[ref16] Eichenbaum H. (2017). Prefrontal–hippocampal interactions in episodic memory. Nat. Rev. Neurosci. 18, 547–558. doi: 10.1038/nrn.2017.74, PMID: 28655882

[ref17] Fan K. H. Francis L. Aslam M. M. Bedison A. Lawrence E. Acharya V.. (2022). Investigation of the independent role of a rare *APOE* variant (L28P; *APOE*4*Pittsburgh) in late-onset Alzheimer disease. Neurobiol. Aging 122, 107–111. doi: 10.1016/j.neurobiolaging.2022.11.007 36528961 PMC9839598

[ref18] Fuster J. M. (2001). The prefrontal cortex—an update. Neuron 30, 319–333. doi: 10.1016/S0896-6273(01)00285-9, PMID: 11394996

[ref19] Gleason C. E. Dowling N. M. Wharton W. Manson J. E. Miller V. M. Atwood C. S.. (2014). Effects of hormone therapy on cognition and mood in recently postmenopausal women: findings from the randomized, controlled KEEPS–cognitive and affective study. PLoS Med. 12, e1001833–e1001825. doi: 10.1371/journal.pmed.1001833, PMID: 26035291 PMC4452757

[ref20] Hao J. Rapp P. R. Leffler A. E. Leffler S. R. Janssen W. G. M. Lou W.. (2006). Estrogen alters spine number and morphology in prefrontal cortex of aged female Rhesus monkeys. J. Neurosci. 26, 2571–2578. doi: 10.1523/JNEUROSCI.3440-05.2006, PMID: 16510735 PMC6793646

[ref21] Harlow S. D. Gass M. Hall J. E. Lobo R. Maki P. Rebar R. W.. (2012). Executive summary of the stages of reproductive aging workshop +10: addressing the unfinished agenda of staging reproductive aging. Climacteric 15, 105–114. doi: 10.3109/13697137.2011.650656, PMID: 22338612 PMC3580996

[ref22] Hu X. Y. Qin S. Lu Y. P. Ravid R. Swaab D. F. Zhou J. N. (2003). Decreased estrogen receptor-α expression in hippocampal neurons in relation to hyperphosphorylated tau in Alzheimer patients. Acta Neuropathol. 106, 213–220. doi: 10.1007/s00401-003-0720-3, PMID: 12819990

[ref23] Ishunina T. A. Fischer D. F. Swaab D. F. (2007). Estrogen receptor α and its splice variants in the hippocampus in aging and Alzheimer’s disease. Neurobiol. Aging 28, 1670–1681. doi: 10.1016/j.neurobiolaging.2006.07.024, PMID: 17010478

[ref24] Kantarci K. Lowe V. J. Lesnick T. G. Tosakulwong N. Bailey K. R. Fields J. A.. (2016a). Early postmenopausal transdermal 17β-estradiol therapy and amyloid-β deposition. J. Alzheimers Dis. 53, 547–556. doi: 10.3233/JAD-160258, PMID: 27163830 PMC4955514

[ref25] Kantarci K. Tosakulwong N. Lesnick T. G. Zuk S. M. Gunter J. L. Gleason C. E.. (2016b). Effects of hormone therapy on brain structure: a randomized controlled trial. Neurology 87, 887–896. doi: 10.1212/WNL.0000000000002970, PMID: 27473135 PMC5035155

[ref26] Kantarci K. Tosakulwong N. Lesnick T. G. Zuk S. M. Lowe V. J. Fields J. A.. (2018). Brain structure and cognition 3 years after the end of an early menopausal hormone therapy trial. Neurology 90, e1404–e1412. doi: 10.1212/WNL.0000000000005325, PMID: 29661902 PMC5902783

[ref27] Karikari T. K. Pascoal T. A. Ashton N. J. Janelidze S. Benedet A. L. Rodriguez J. L.. (2020). Blood phosphorylated tau 181 as a biomarker for Alzheimer’s disease: a diagnostic performance and prediction modelling study using data from four prospective cohorts. Lancet Neurol. 19, 422–433. doi: 10.1016/S1474-4422(20)30071-5, PMID: 32333900

[ref28] Kelly J. F. Bienias J. L. Shah A. Meeke K. A. Schneider J. A. Soriano E.. (2008). Levels of estrogen receptors α and β in frontal cortex of patients with Alzheimer’s disease: relationship to Mini-mental state examination scores. Curr. Alzheimer Res. 5, 45–51. doi: 10.2174/156720508783884611, PMID: 18288931 PMC3268687

[ref29] Kim C. Harlow S. D. Zheng H. McConnell D. S. Randolph J. F. (2017). Changes in androstenedione, dehydroepiandrosterone, testosterone, estradiol, and estrone over the menopausal transition. Women’s Midlife Health 3:9. doi: 10.1186/s40695-017-0028-4, PMID: 29333273 PMC5761074

[ref30] Kim J. Y. Mo H. Kim J. Kim J. W. Nam Y. Rim Y. A.. (2022). Mitigating effect of estrogen in Alzheimer’s disease-mimicking cerebral organoid. Front. Neurosci. 16:816174. doi: 10.3389/fnins.2022.816174, PMID: 35401074 PMC8990972

[ref31] Koran M. E. I. Wagener M. Hohman T. J. for the Alzheimer’s Neuroimaging Initiative (2017). Sex differences in the association between AD biomarkers and cognitive decline. Brain Imaging Behav. 11, 205–213. doi: 10.1007/s11682-016-9523-8, PMID: 26843008 PMC4972701

[ref33] Kunzler J. Youmans K. L. Yu C. LaDu M. J. Tai L. M. (2014). APOE modulates the effect of estrogen therapy on Aβ accumulation EFAD-Tg mice. Neurosci. Lett. 560, 131–136. doi: 10.1016/j.neulet.2013.12.032, PMID: 24368217 PMC3955876

[ref34] Li Z. Shue F. Zhao N. Shinohara M. Bu G. (2020). APOE2: protective mechanism and therapeutic implications for Alzheimer’s disease. Mol. Neurodegener. 15:63. doi: 10.1186/s13024-020-00413-4, PMID: 33148290 PMC7640652

[ref001] Mowinckel A. M. Vidal-Piñeiro D. (2019). Visualisation of nrain statistics with R-packages ggseg and ggseg3d. arXiv:1912.08200.

[ref35] Nasreddine Z. S. Phillips N. A. Bédirian V. Charbonneau S. Whitehead V. Collin I.. (2005). The Montreal cognitive assessment, MoCA: a brief screening tool for mild cognitive impairment. J. Am. Ger. Soc. 53, 695–699. doi: 10.1111/j.1532-5415.2005.53221.x, PMID: 15817019

[ref36] Nelson R. E. Grebe S. K. O’Kane D. J. Singh R. J. (2004). Liquid chromatography–tandem mass spectrometry assay for simultaneous measurement of estradiol and Estrone in human plasma. Clin. Chem. 50, 373–384. doi: 10.1373/clinchem.2003.025478 14656902

[ref37] Neu S. C. Pa J. Kukull W. Beekly D. Kuzma A. Gangadharan P.. (2017). Apolipoprotein E genotype and sex risk factors for Alzheimer’s disease: a meta-analysis. JAMA Neurol. 74, 1178–1189. doi: 10.1001/jamaneurol.2017.2188, PMID: 28846757 PMC5759346

[ref38] Newhouse P. Dumas J. (2015). Estrogen-cholinergic interactions: implications for cognitive aging. Horm. Behav. 74, 173–185. doi: 10.1016/j.yhbeh.2015.06.022, PMID: 26187712 PMC4573353

[ref39] Nilsen J. Chen S. Irwin R. W. Iwamoto S. Brinton R. D. (2006). Estrogen protects neuronal cells from amyloid beta-induced apoptosis via regulation of mitochondrial proteins and function. BMC Neurosci. 7, 1–14. doi: 10.1186/1471-2202-7-74, PMID: 17083736 PMC1636062

[ref01] Nyberg L. (2016). Functional brain imaging of episodic memory decline in aging. J. Intern. Med. 281, 65–74. 27453565 10.1111/joim.12533

[ref40] Österlund M. K. Gustafsson J. A. Keller E. Hurd Y. L. (2000). Estrogen receptor β (ERβ) messenger ribonucleic acid (mRNA) expression within the human forebrain: distinct distribution pattern to ERα mRNA1. J. Clin. Endocrinol. Metab. 85, 3840–3846. doi: 10.1210/jcem.85.10.6913 11061547

[ref41] Osterlund M. K. Hurd Y. L. (2001). Estrogen receptors in the human forebrain and the relation to neuropsychiatric disorders. Prog. Neurobiol. 64, 251–267. doi: 10.1016/s0301-0082(00)00059-9, PMID: 11240308

[ref42] Ottowitz W. E. Siedlecki K. L. Lindquist M. A. Dougherty D. D. Fischman A. J. Hall J. E. (2008). Evaluation of prefrontal–hippocampal effective connectivity following 24 hours of estrogen infusion: an FDG-PET study. Psychoneuroendocrino. 33, 1419–1425. doi: 10.1016/j.psyneuen.2008.09.013, PMID: 18977091 PMC2633466

[ref43] Pérez-GrijalbaV.RomeroJ.PesiniP.SarasaL.MonleónI.San-JoséI.. (2019). Plasma Ab42/40 ratio detects early stages of Alzheimer’s disease and correlates with CSF and neuroimaging biomarkers in the AB255 Study. J. Prev. Alz. Dis. 1–8. doi: 10.14283/jpad.2018.4130569084

[ref44] Phillips S. M. Sherwin B. B. (1992). Effects of estrogen on memory function in surgically menopausal women. Psychoneuroendocrinology 17, 485–495. doi: 10.1016/0306-4530(92)90007-T 1484915

[ref45] R Core Team (2023). R: A language and environment for statistical computing. Vienna, Austria: R Foundation for Statistical Computing.

[ref46] Randolph J. F. Zheng H. Sowers M. R. Crandall C. Crawford S. Gold E. B.. (2011). Change in follicle-stimulating hormone and estradiol across the menopausal transition: effect of age at the final menstrual period. J Clin Endocr Metab. 96, 746–754. doi: 10.1210/jc.2010-1746, PMID: 21159842 PMC3047231

[ref47] Rapp P. R. Morrison J. H. Roberts J. A. (2003). Cyclic estrogen replacement improves cognitive function in aged Ovariectomized Rhesus monkeys. J. Neurosci. 23, 5708–5714. doi: 10.1523/JNEUROSCI.23-13-05708.2003, PMID: 12843274 PMC6741262

[ref48] Resnick S. M. Espeland M. A. Jaramillo S. A. Hirsch C. Stefanick M. L. Murray A. M.. (2009). Postmenopausal hormone therapy and regional brain volumes: the WHIMS-MRI study. Neurology 72, 135–142. doi: 10.1212/01.wnl.0000339037.76336.cf, PMID: 19139364 PMC2677493

[ref49] Saleh R. N. Hornberger M. Ritchie C. W. Minihane A. M. (2023). Hormone replacement therapy is associated with improved cognition and larger brain volumes in at-risk APOE4 women: results from the European prevention of Alzheimer’s disease (EPAD) cohort. Alzheimers Res. Ther. 15:10. doi: 10.1186/s13195-022-01121-5, PMID: 36624497 PMC9830747

[ref50] Sampedro F. Vilaplana E. De Leon M. J. Alcolea D. Pegueroles J. Montal V.. (2015). *APOE*-by-sex interactions on brain structure and metabolism in healthy elderly controls. Oncotarget 6, 26663–26674. doi: 10.18632/oncotarget.5185, PMID: 26397226 PMC4694943

[ref51] Sundermann E. E. Tran M. Maki P. M. Bondi M. W. (2018). Sex differences in the association between apolipoprotein E ε4 allele and Alzheimer's disease markers. Alzheimers Dement. 10, 438–447. doi: 10.1016/j.dadm.2018.06.004, PMID: 30182053 PMC6120724

[ref52] Taxier L. R. Philippi S. M. Fleischer A. W. York J. M. LaDu M. J. Frick K. M. (2022). APOE4 homozygote females are resistant to the beneficial effects of 17β-estradiol on memory and CA1 dendritic spine density in the EFAD mouse model of Alzheimer’s disease. Neurobiol. Aging 118, 13–24. doi: 10.1016/j.neurobiolaging.2022.06.005, PMID: 35843109 PMC10756028

[ref53] Tepper P. G. Randolph J. F. McConnell D. S. Crawford S. L. El Khoudary S. R. Joffe H.. (2012). Trajectory clustering of estradiol and follicle-stimulating hormone during the menopausal transition among women in the study of Women’s health across the nation (SWAN). J Clin Endocr Metab. 97, 2872–2880. doi: 10.1210/jc.2012-1422, PMID: 22659249 PMC3410268

[ref54] Testo A. A. Makarewicz J. McGee E. Dumas J. A. (2024). Estradiol associations with brain functional connectivity in postmenopausal women. Menopause 31, 218–224. doi: 10.1097/GME.0000000000002321, PMID: 38385731 PMC10885742

[ref55] Thurston R. C. Chang Y. Barinas-Mitchell E. Jennings J. R. Landsittel D. P. Santoro N.. (2016). Menopausal hot flashes and carotid intima media thickness among midlife women. Stroke 47, 2910–2915. doi: 10.1161/STROKEAHA.116.014674, PMID: 27834746 PMC5134903

[ref56] Thurston R. C. Wu M. Chang Y. F. Aizenstein H. J. Derby C. A. Barinas-Mitchell E. A.. (2023). Menopausal vasomotor symptoms and white matter hyperintensities in midlife women. Neurology 100, e133–e141. doi: 10.1212/WNL.0000000000201401, PMID: 36224031 PMC9841446

[ref57] Valencia-Olvera A. C. Weng J. M. Christensen A. LaDu M. J. Pike C. J. (2023). Role of estrogen in women’s Alzheimer’s disease risk as modified by *APOE*. J. Neuroendocrinol. 35:e13209. doi: 10.1111/jne.13209, PMID: 36420620 PMC10049970

[ref58] Xu H. Gouras G. K. Greenfield J. P. Vincent B. Naslund J. Mazzarelli L.. (1998). Estrogen reduces neuronal generation of Alzheimer β-amyloid peptides. Nature Med. 4, 447–451. doi: 10.1038/nm0498-447 9546791

[ref59] Yue Y. Hu L. Tian Q. Jiang J. Dong Y. Jin Z.. (2007). Effects of long-term, low-dose sex hormone replacement therapy on hippocampus and cognition of postmenopausal women of different apoE genotypes. Acta Pharmacol. Sin. 28, 1129–1135. doi: 10.1111/j.1745-7254.2007.00618.x, PMID: 17640473

[ref60] Zeydan B. Tosakulwong N. Schwarz C. G. Senjem M. L. Gunter J. L. Reid R. I.. (2019). Association of bilateral Salpingo-oophorectomy before menopause onset with medial temporal lobe neurodegeneration. JAMA Neurol. 76, 95–100. doi: 10.1001/jamaneurol.2018.3057 30326011 PMC6439881

[ref61] Zsido R. G. Williams A. N. Barth C. Serio B. Kurth L. Mildner T.. (2023). Ultra-high-field 7T MRI reveals changes in human medial temporal lobe volume in female adults during menstrual cycle. Nat. Mental Health 1, 761–771. doi: 10.1038/s44220-023-00125-w

